# The Genetic Factors Controlling the Accumulation of Amylase/Trypsin Inhibitors (ATIs) in Barley for Enhancing Human Nutrition and Health

**DOI:** 10.1002/fsn3.70990

**Published:** 2026-01-10

**Authors:** Dalia Z. Alomari, Samar G. Thabet, Mashael D. Alqahtani, Matías Schierenbeck, Sarah Joestl, Sabrina Geisslitz, Katharina A. Scherf, Andreas Börner, Ahmad M. Alqudah

**Affiliations:** ^1^ Department of Clinical Nutrition and Dietetics, Faculty of Applied Medical Sciences The Hashemite University Zarqa Jordan; ^2^ Department of Botany, Faculty of Science Fayoum University Fayoum Egypt; ^3^ Department of Biology, College of Sciences Princess Nourah Bint Abdulrahman University Riyadh Saudi Arabia; ^4^ Physiology and Cell Biology Department Leibniz Institute of Plant Genetics and Crop Plant Research (IPK), OT Gatersleben Seeland Germany; ^5^ Leibniz Institute for Food Systems Biology at the Technical University of Munich Freising Germany; ^6^ Department of Bioactive and Functional Food Chemistry, Institute of Applied Biosciences Karlsruhe Institute of Technology (KIT) Karlsruhe Germany; ^7^ Technical University of Munich, School of Life Sciences, Professorship of Food Biopolymer Systems TUM Freising Germany; ^8^ Genebank Department Leibniz Institute of Plant Genetics and Crop Plant Research (IPK), OT Gatersleben Seeland Germany; ^9^ Biological Science Program, Department of Biological and Environmental Sciences, College of Arts and Sciences Qatar University Doha Qatar

**Keywords:** amylase/trypsin inhibitors, barley‐based food, human nutrition and health

## Abstract

Amylase trypsin inhibitors (ATIs) are a group of proteins in all cereals' seeds, including wheat, barley, rye, and maize. Currently, ATIs are the most studied wheat components since they are linked to celiac disease and baker's asthma. The interest has increased since 2012, when they were shown to trigger the innate immune system and intestinal and extra‐intestinal inflammation. Even though ATIs are present in many plant‐based diets, the genetic factors underlying these proteins have not yet been investigated in barley. To this end, this study was designed to measure ten ATI proteins in a diverse barley collection grown under field conditions. Ample natural variation among the accessions in ten ATIs has been measured, which showed a substantial role in the accumulation of ATI_total, such as CMa, CMd, CMe, BTI_CMc, and AIBDAI_1. A genome‐wide association scan (GWAS) utilizing a large number of molecular markers demonstrated that the accumulation of ATIs was influenced by many small to medium quantitative trait nucleotides (QTNs). Eight QTNs showed the highest association with ATIs, particularly AIBDAI, whereas these QTNs negatively influenced ATI accumulation. Genomic investigations identified Serine/threonine protein phosphatase as a putative candidate gene. Our research provides the initial analysis of the ATI proteins found in barley, which might potentially contribute to enhancing the quality of barley‐based food products. As a result, the study demonstrated that cultivars with lower ATI proteins can speed up their integration to improve the quality of barley products and diminish the possibility of some diseases.

## Graphical abstract

ATIs in cereal seeds link to celiac disease, asthma, and immune response. Here, we report the first study exploring genetic factors affecting ATI levels in barley. GWAS identified multiple QTNs influencing ATI accumulation with notable variation. Eight QTNs are associated with lower ATI levels; a key gene was identified.

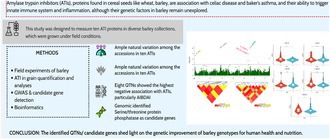

**Abbreviations**
AI: amylase‐inhibitors
ANOVA: analysis of variance
ASI: amylase/subtilisin‐inhibitor
ATI: amylase/trypsin‐inhibitor
BDAI: barley dimeric amylase inhibitor
BMAI: barley monomeric amylase inhibitor
CAA: chloroacetamide
CD: celiac disease
CE: collision energies
CI: chymotrypsin‐inhibitors
CM: chloroform/methanol
GWAS: genome‐wide association scan
QTL: quantitative trait loci
QTN: quantitative trait nucleotides
SCI‐1A: subtilisin/chymotrypsin‐inhibitor 1A
SCI‐1B: subtilisin/chymotrypsin‐inhibitor 1B
SIDA: stable isotope dilution analysis
SNP: single‐nucleotide polymorphism
SRM: selected reaction monitoring
TCEP: Tris(2‐carboxyethyl) phosphine

## 1 Introduction

Barley ( *Hordeum vulgare* L.) is a member of the *Poaceae* family and ranked as the fourth most‐produced cereal, with a global output of 151.6 million metric tons in 2022, according to FAOSTAT (FAO 2022). Its production has been relatively consistent in recent years as it is more tolerant to climate changes and environmental stresses than other relative cereal crops like wheat. Many aromatic and flavored food items known for their bioactive phenolic compounds, unique taste, and health benefits have traditionally been produced from barley flour (Nakov et al. 2022). Due to its high nutritional enrichment values, barley flour has been considered an excellent source of natural α‐ and β‐amylase enzymes (Padilla‐Torres et al. 2022). Various odors and flavors forms (Rogner et al. 2021), in addition to high dietary fiber and β‐glucan content, make it effective in preventing degenerative illnesses (Farag et al. 2022). Moreover, barley is acknowledged for its high content of carbohydrates, vitamins, minerals, and protein. Consequently, it is considered a valuable dietary additive for creating various health‐enhancing, nutritious, and functional food items (Farag et al. 2022). Therefore, barley flour showed health evidence that it should be used instead of wheat flour, which can enhance enriched loaves' technical, rheological, physicochemical, and organoleptic characteristics (Aslam et al. 2023).

Amylase trypsin inhibitors (ATIs) are a class of proteins present in many plant‐based diets. ATIs comprise 2%–4% of the wheat grain proteins (Simonetti, Bosi, Negri, and Dinelli 2022). These proteins contribute to the plant's innate defense against pests and diseases (Carbonero and García‐Olmedo 1999). Regarding human ingestion, ATIs can have many effects, which can be either beneficial or detrimental, depending on an individual's health condition and dietary habits (Zevallos et al. 2017; Zevallos et al. 2019). These proteins hinder the function of amylase and trypsin, digestive enzymes that break down carbohydrates and proteins (Simonetti, Bosi, Negri, and Dinelli 2022). In wheat, ATIs can decrease the pace of digestion and absorption of nutrients by blocking these enzymes that might have an impact on stimulating specific types of immune cells in the intestines by activating myeloid cells (Zevallos et al. 2017). ATI's biological activity characterization had been validated using a disease experiment that employed TLR4‐responsive mice and human cells, resulting in severe inflammatory bowel disease (Zevallos et al. 2017), fatty liver disease (Ashfaq‐Khan et al. 2019), food and respiratory allergies (Zevallos et al. 2019), and Alzheimer's disease (dos Santos Guilherme et al. 2020). In addition, ATIs are known to cause respiratory and food allergies that are classical and immediate‐type IgE‐mediated (Kalunke et al. 2020). Additionally, ATIs are a significant cause of irritable bowel syndrome, which affects up to 15% of societies (Fritscher‐Ravens et al. 2019). All three subfamilies of wheat ATIs are non‐gluten protein fractions. Gluten has been the primary focus of research on wheat protein families related to digestive health (Caminero and Verdu 2019). Gluten plays a crucial role in determining the quality of bread and pasta. However, it also triggers celiac disease (Caminero and Verdu 2019). ATIs, unlike gluten, have recently become the focus of clinical research in wheat; most previous studies aimed to characterize the ATIs in cereals, especially in grain (Franco et al. 2000). Recent review papers provided detailed explanations about the features and importance of ATIs in wheat (Aslam et al. 2023; Geisslitz et al. 2021; Simonetti, Bosi, Negri, and Dinelli 2022). Few studies have been done on ATI's characterization in barley (Jones and Fontanini 2003; Lazaro et al. 1988; Terras et al. 1993). Consequently, it is essential to comprehend the ATIs in barley as a nutritional resource.

The diversity of the ATI proteins is vast, with a reported count of up to 90 proteins. However, only about 20 proteins have undergone thorough research and classification based on their mono, di, or tetrameric structure (Geisslitz et al. 2021; Weegels and America 2023). There were a few attempts to understand the variation of ATI activation in different levels of ploidy and release years based on their ATI gene sequences (Simonetti, Bosi, Negri, Baffoni, et al. 2022). Low ATI activation in einkorn can be attributed to potential mutations in the associated coding genes (Franco et al. 2000). Recently, genotype by environment interaction influences the variation of ATI inhibitory properties in wheat and human health and nutrition (Simonetti, Bosi, Negri, and Dinelli 2022). The phylogenetic analysis based on amino acid sequences showed that the monomeric and dimeric forms are grouped but separate from the tetrameric subunits and trypsin inhibitors (Geisslitz et al. 2021).

The sequence analysis of the wheat genome indicates that the B and D genomes encode the majority of ATIs (Juhasz et al. 2018). In wheat, the short arms of chromosomes 6B and 6D house the genes encoding the monomeric form, while chromosomes 3B and 3D house the genes encoding the dimeric form (Geisslitz et al. 2021), whereas tetrameric genes, on the other hand, are located on chromosomes 4B, 4D, 7B, and 7D. Genes on the group 4 chromosomes of all three genomes encode the trypsin inhibitor CMX. Multiple sequence alignments on the wheat reference genome significantly enhanced the gene locations (Bose et al. 2020). Recently, a genome‐wide association mapping technique employing 22,220 Diversity Arrays Technology (DArT) markers in 149 ancient and contemporary bread wheat cultivars cultivated at three distinct field sites was conducted to define the genetic architecture controlling ATI proteins in wheat (El Hassouni et al. 2021). The analysis identified an intricate genetic structure consisting of several quantitative trait loci (QTL), of which the strongest QTLs were identified on chromosome 3B for ATI 0.19‐like and on chromosome 6B for ATI 0.28, which accounted for ~70% of the genotypic variation (El Hassouni et al. 2021).

Most of our understanding of ATIs in barley is based on research conducted on wheat that emphasizes the significance of studying ATIs in barley. Moreover, to our knowledge, very few old studies on ATIs in barley (Jones and Fontanini 2003; Lazaro et al. 1988; Terras et al. 1993) and the genetic factors underlying these proteins have not yet to be investigated. Here, we provided the first comprehensive evaluation of ATIs in barley. Therefore, we used a diverse spring barley collection (EcoSeed) of 184 accessions worldwide as the first study. We applied GWAS utilizing a set of > 125 K SNPs, which was the basis for the underlying genetic control of the ATIs in barley, and further bioinformatics analyses to detect the potential candidate genes regulating the expression of ATIs.

## 2 Materials and Methods

### 2.1 Plant Material and Field Experiments

In the current study, we used the ‘EcoSeed collection’ of barley, consisting of 184 diverse spring barley accessions, as reported before by Ahmad M. Alqudah et al. (2021). Based on their seed quality, the collection was selected from the 23,800 accessions stored at the Genebank, IPK‐Gatersleben, Germany. We cultivated the collection on the experimental field at the Leibniz Institute of Plant Genetics and Crop Plant Research (IPK) in Gatersleben, Germany (11°16′LE; 51°49′LN) during the spring 2018 growing season. Two hundred seeds were directly sown into clay loam soil for each accession as a randomized block design. Three 1 m^2^ plots for each accession had been grown, and we spaced the plots by two rows within each plot. We managed the plots using conventional local agronomic practices.

The ten ATIs were measured using the quantitation method described by Joestl et al. (2025). For the population used in the current study, UltiMate 3000 HPLC (Dionex, Idstein, Germany) with an Aqua‐C18 column (Phenomenex, Aschaffenburg, Germany) coupled to a Triple Quadrupole mass spectrometer (MS, Thermo Fisher Scientific, Bremen, Germany) was used, as extensively explained by Joestl et al. (2025). Detailed methods of ATIs quantitation can be found in the Data S1.

### 2.2 Genotyping and Population Stratification Analyses

For each of the 184 accessions, we combined the leaf material from 10 seedlings at the three‐leaf stage for DNA extraction. Trait Genetics GmbH, located in Gatersleben, Germany, genotyped the collection using the 9 K Illumina Infinium iSELECT Genotyping BeadChip www.traitgenetics.com. Finally, the chip produced 7865 single‐nucleotide polymorphisms (SNPs). We also used the genotyping‐by‐sequencing (GBS) method to genotype the EcoSeed collection. The SNP underwent a quality control and filtration procedure where a minor allele frequency of ≥ 5% was applied according to the procedure previously described (Ahmad M Alqudah et al. 2019). After completing quality tests, we acquired 122,213 SNPs out of 233,095 SNPs. In total, 127,022 polymorphic SNPs were physically anchored and used in the GWAS analyses, as previously described by Ahmad M. Alqudah et al. (2021). The population structure was determined by implementing a principal component analysis, with the kinship matrix among the accessions also being considered (Ahmad M Alqudah et al. 2019; Alqudah et al. 2021).

We generated genome‐wide pairwise estimates of linkage disequilibrium (LD) across the whole genome using the squared correlation (*r* ^2^) between pairs of polymorphic SNPs. The LD and LD decay patterns were calculated as *r* ^2^ against the distance between pairs of polymorphic SNPs (in megabases, Mbp) published (Alqudah et al. 2021).

### 2.3 Phenotypic Data Analyses

We conducted a statistical analysis using different R packages to identify a significant relationship between the traits; *p* ≤ 0.05 was determined using Pearson correlation analysis, with the corrplot function from the R package corrplot employed for this purpose (Wei et al. 2021). We analyzed the phenotypic data using residual maximum likelihood in mixed linear models. We determined the phenotypic means of each trait for each accession using the best linear unbiased estimates (BLUEs) from the lme4 package (Bates et al. 2015), as detailed in Alqudah et al. (2019). We ultimately computed each variable's broad‐sense heritability (*H* ^2^) by dividing the genetic variation by the phenotypic variance, including the error variance described by Alqudah et al. (2021). We used the SRplot as a platform for data visualization and graphing (Tang et al. 2023).

### 2.4 Candidate Gene Detection Approach

We conducted the GWAS by applying the FarmCPU model in the rMVP R package (Yin et al. 2021). The FarmCPU model was used to reduce overfitting, which was explained by a reduction in false negatives and false positives associations. The model was successfully applied in other populations and studied. We computed the false discovery rate (FDR) for each trait individually. We utilized only the association signals above the FDR threshold of 0.001 (−log_10_ *p*‐values > FDR) for further analyses, as explained by Ahmad M. Alqudah et al. (2019). We created Manhattan plots for each measured characteristic to illustrate the associations between markers and traits on the seven barley chromosomes.

We used the released barley genome sequence to align strongly related markers based on their physical location (Mascher et al. 2017) after they passed the false discovery rate (FDR) threshold. We considered the spatial location of these specific SNPs and confirmed clusters of SNPs to be in linkage disequilibrium. The single associated SNP with ATI traits was mentioned as a quantitative trait nucleotide (QTN). We chose only genes with a high level of confidence that include the corresponding SNPs (Mascher et al. 2017) with their annotation in the latest version of the BARLEX database (http://apex.ipk‐gatersleben.de/apex/f?p=284:10). Subsequently, we verified the placement of the SNPs and clarified whether they fell into the introns or the exons. We used the single‐nucleotide polymorphisms (SNPs) within the candidate gene's physical site to validate expression assessments using data from the BARLEX database.

## 3 Results

### 3.1 Natural Variations of α‐Amylase/Trypsin Inhibitors Concentration in the EcoSeed Spring Barley Collection

**FIGURE 1:**
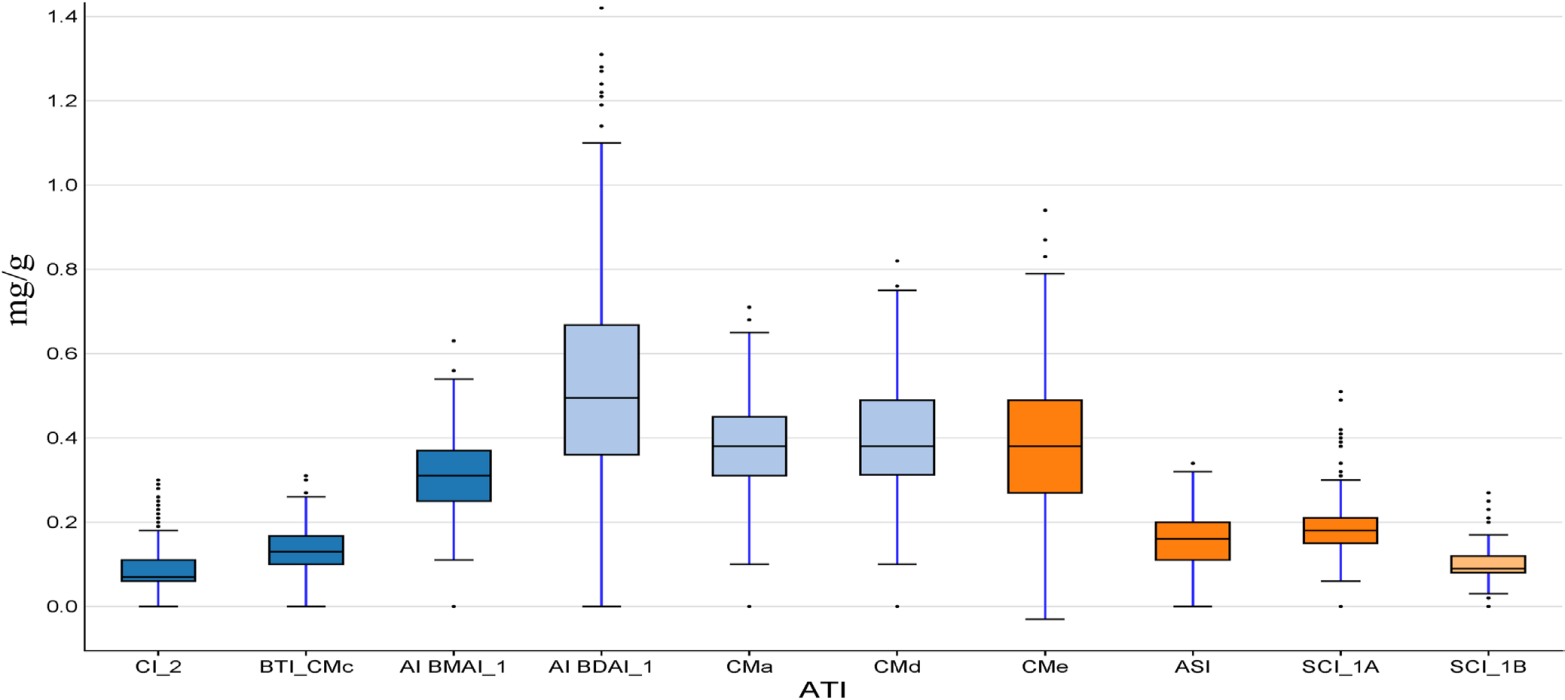
Boxplots showing the concentration (mg/g) of different amylase trypsin inhibitors (ATIs) in 184 spring barley accessions.

**FIGURE 2:**
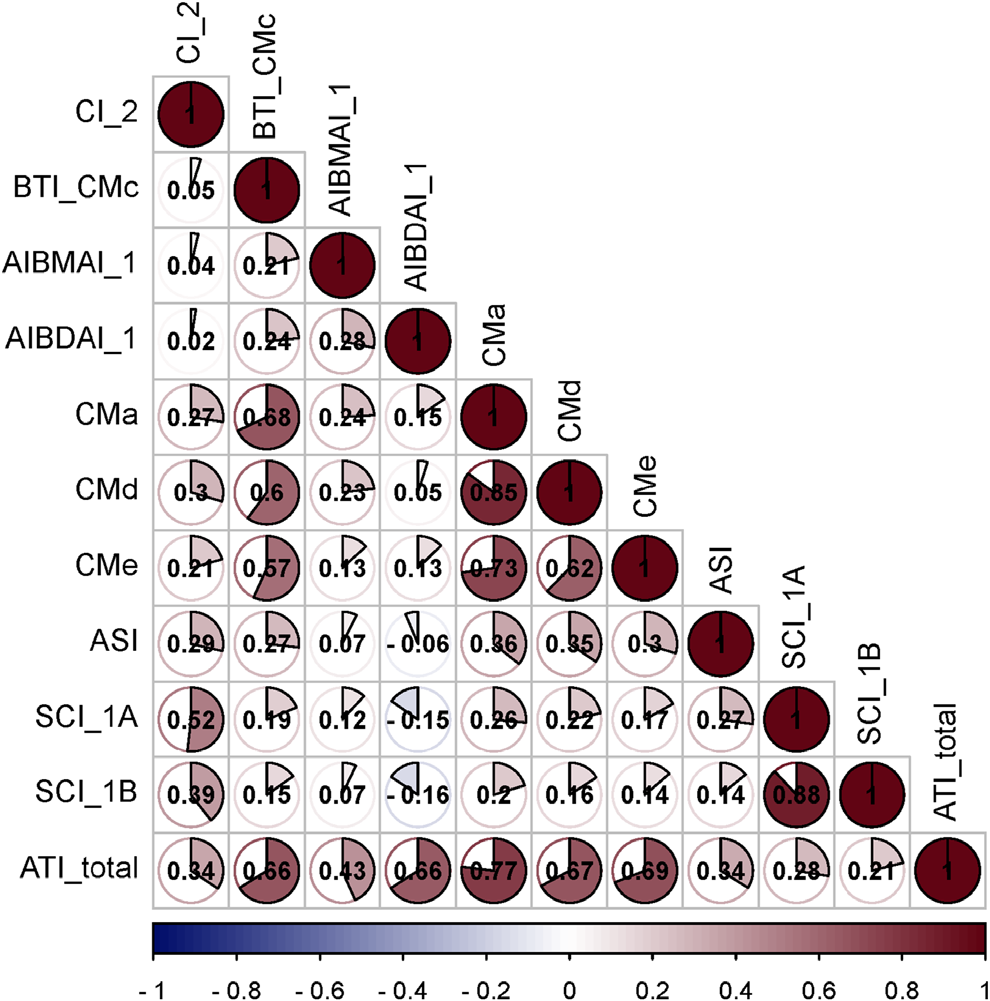
Correlation among amylase trypsin inhibitors (ATIs) in 184 spring barley genotypes. The degree of significance for all correlations across different years was *p* < 0.001. The color reflects the strength of the correlation.

The box plots in Figure 1 indicate the data variability (mg/g) for the different ATI proteins in the barley collection evaluated and grown under field conditions. As a means, the proteins exhibiting the highest values were AI BDA_1 (0.655 mg/g), CMa (0.395 mg/g), CMd (0.408 mg/g), CMe (0.398 mg/g), and AI_BMAI_1 (0.317 mg/g). The rest of the proteins (SCI_1A, ASI, SCI_1B, BTI_CMc, and CI_2) showed lower values that ranged between 0.09 and 0.2 mg/g (Figure 1). Summary statistics are shown in Table S1, demonstrating the high variation in ATI proteins among the barley accessions. However, the high coefficients of variation values reported for ATIs indicate that they are suitable for examining phenotypic/genetic variation, which can be attributed to the variety of germplasm (geographical origins) and/or the status of germplasm (cultivar and landrace). A summary of the ten ATIs revealed a 230% difference in total ATI content across the barley collection, with the six‐rowed landrace ‘HOR 2571’ exhibiting the lowest value (1.572 mg/g) and the two‐rowed landrace ‘HOR 2202’ demonstrating the highest (5.199 mg/g) (Figure 1 and Figure S1). The correlation coefficients at *p* < 0.001 among the ten ATI proteins evaluated exhibited a range of values from −0.16 to 0.85 (Figure 2). Significant positive correlations at *p* < 0.001 were observed between ATI_total and CMa, CMd, CMe, BTI_CMc, and AI BDAI_1. BTI_CMc exhibited a high correlation with CMa, CMb, and CMe. SCI_1A and SCI_1B were also found to be highly correlated. For their part, CI_2 presents positive correlations with SCI_1A, SCI_1B, CMa, CMd, CMe, and ASI. Furthermore, a negative association was observed between AI BMAI_1 and SCI_1A/SCI_1B at *p* < 0.001 (Figure 2). Extensive ATI analyses in this barley population are published by Joestl et al. (2025).

**FIGURE 3:**
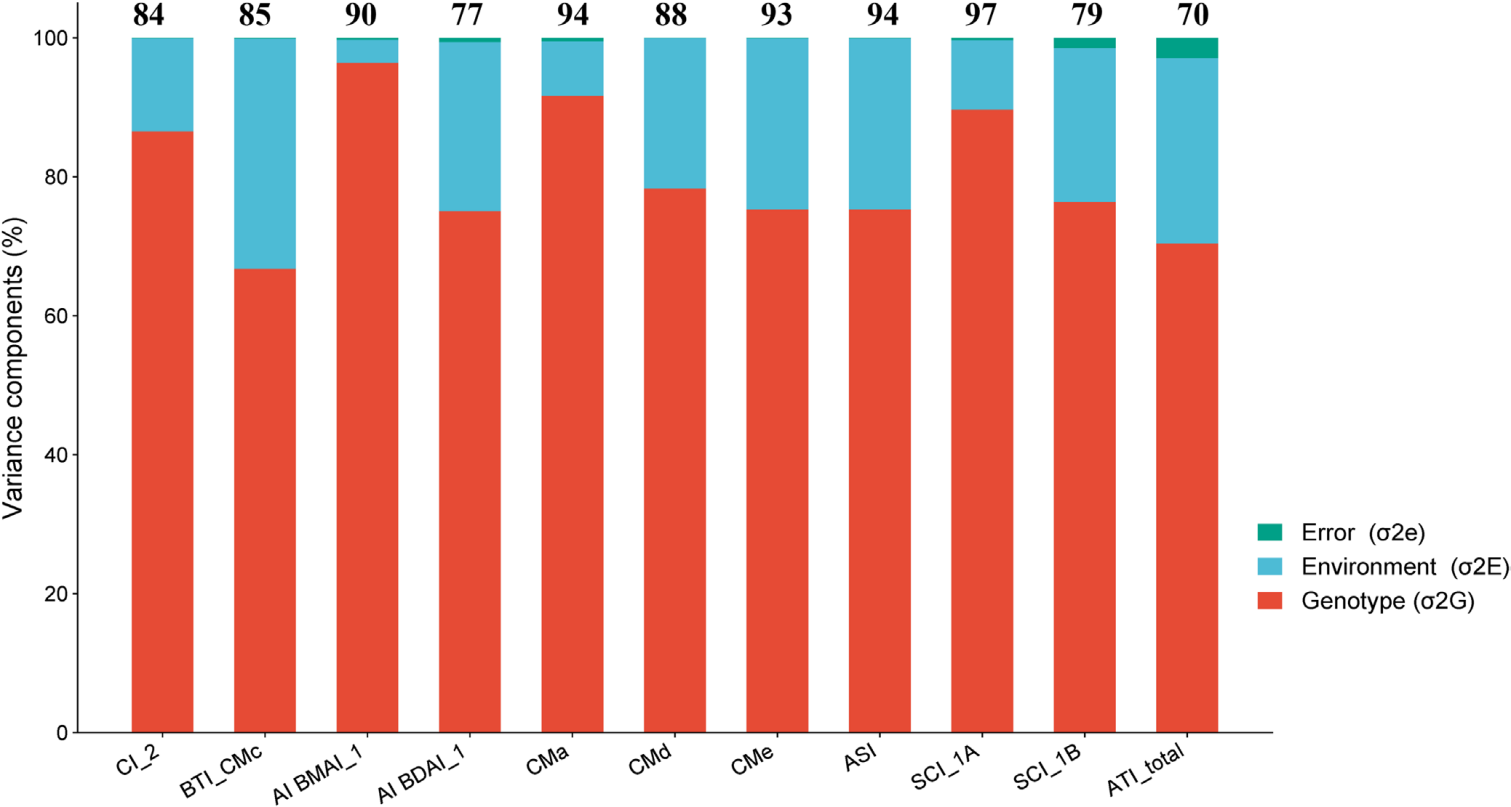
Variance component analysis and broad‐sense heritability of different amylase trypsin inhibitors (ATIs) in 184 spring barley accessions. Bold letters in each bar indicate the genotypic variability.

Broad‐sense heritability (*H* ^2^) of ATI proteins ranging from 0.70 to 0.97 was obtained for each trait (Figure 3). Most traits exhibited high heritability values (0.84–0.94), whereas AIBDAI_1, SCI_1B, and ATI_total presented intermediate values (0.70–0.79). These findings indicate that the EcoSeed collection has been appropriately selected for having high phenotypic variation among accessions with a low environmental influence (Figure 3). It suggests that ATIs are primarily determined by genetic factors and offer a potential and exciting new chance to study the genetics of ATI proteins, thereby creating a fresh avenue for research.

### 3.2 QTNs and Genetic Regions Associated With α‐Amylase/Trypsin Inhibitors in a Global Spring Barley Collection

The analyses detected significant 444 QTNs (−log10 > FDR; *p* < 0.0001) distributed over the seven barley chromosomes associated with different ATIs (Table S2). In terms of QTN numbers per chromosome, chromosomes 3H harbor 194 QTNs, 4H (94 QTNs), 7H (78 QTNs), and 5H (39 QTNs) reported the highest number of QTNs with these traits. In comparison, 6H (23 QTNs), 2H (9 QTNs), and 1H (7 QTNs) showed a lower number (Table S2 and Figures S2–S6). Overall, eight significant QTNs were reported for AI.BDAI on chromosomes 1H (1), 3H (1), 5H (3), 6H (2), and 7H (1) with −log_10_ ranging 4.64–10.15. For AI.BMAI, 14 QTNs with −log_10_ ranging 4.40–6.04 were identified on 1H (3), 2H (3), 3H (1), 4H (4), 5H (1), and 7H (2). For their part, 124 QTNs related to ASI were reported on chromosomes 2H (1), 3H (12), 4H (80), 5H (23), and 7H (8), showing −log_10_ of 4.87–6.64. Six QTNs were reported for ATI_total on 3H (2), 5H (1), and 6H (3) with −log_10_ of 4.82–8.78. On chromosomes 1H, 2H (2), 3H (2), and 7H we detected 6 QTNs related to BTI_CMc (−log_10_ 6.08–7.31), while 23 QTNs were spotted for CI_2 on 1H, 2H, 3H (2), 4 (8), 6H (4), and 7H (7) (−log_10_ 4.41–5.79). A total of 245 marker‐traits associations were reported for CMa on 3H (171 QTNs), 5H (8), 6H (13), and 7H (53) (−log_10_ 4.41–6.92), whereas only 3 QTNs were detected for CMd on 3H (2) and 7H (−log_10_ 4.84–6.08). For CMe, 7 QTNs were reported on chromosomes 1H, 2H, 3H, 4H (2), 5H, and 6H (−log_10_ 4.84–7.01), while eight significant associations were reported for SCI_1A on 2H, 5H (2), and 7H (5) (−log_10_ 4.43–5.21). The effects (mg/g) of these 444 QTNs ranged between −0.24 and +0.16 for AI.BDAI; −0.076 to +0.061 for AI.BMAI; −0.058 to +0.035 for ASI; −0.44 to +0.291 for ATI_total; +0.0197 to +0.057 for BTI_CMc; −0.019 to +0.015 for CI_2; −0.0756 to +0.0536 for CMa; −0.0567 to +0.064 for CMd; −0.0589 to +0.0916 for CMe; and −0.0459 to +0.0697 for SCI_1A (Table S2). More details about the QTNs detected, such as chromosome, marker position, effect, −log_10_, target, and alternative alleles, are displayed in Table S2. According to the significance of QTNs, the eight most significant QTNs with −log10 ≥ seven were associated with AI.BDAI (5 QTNs) and one QTN for ATI_Total, BTI_CMc, and CMe (Table 1). Interestingly, these QTNs harm the proteins, a crucial factor in diminishing the inhibitor. These outputs might be valuable for selecting accessions and expanding our knowledge of the genetic pathways contributing to the accumulation of ATIs.

**TABLE 1:** List of ATIs isoforms and high‐associated SNPs/QTNs with their physical position based on Morex (v3) genome pseudomolecule.

ATI_trait	SNP name	Chr.	POS	REF	ALT	Effect
AI. BDAI	m_842	1	24,731,616	T	C	−0.168469803
BTI_CMc	m_20328	2	54,978,556	C	G	0.027957059
AI. BDAI	m_31050	3	255,737,083	G	A	−0.172496814
CMe	BOPA2_12_30554	4	618,415,678	A	C	−0.04208877
AI. BDAI	m_70996	5	549,651,999	G	A	−0.246685543
AI. BDAI	SCRI_RS_207000	5	620,734,830	A	G	−0.07922371
AI. BDAI	m_89998	6	5,231,873	G	A	−0.209608007
ATI_total	m_90200	6	104,925,337	G	A	−0.44013511

### 3.3 Candidate Genes Underlying ATI Protein Accumulation in Barley

**FIGURE 4:**
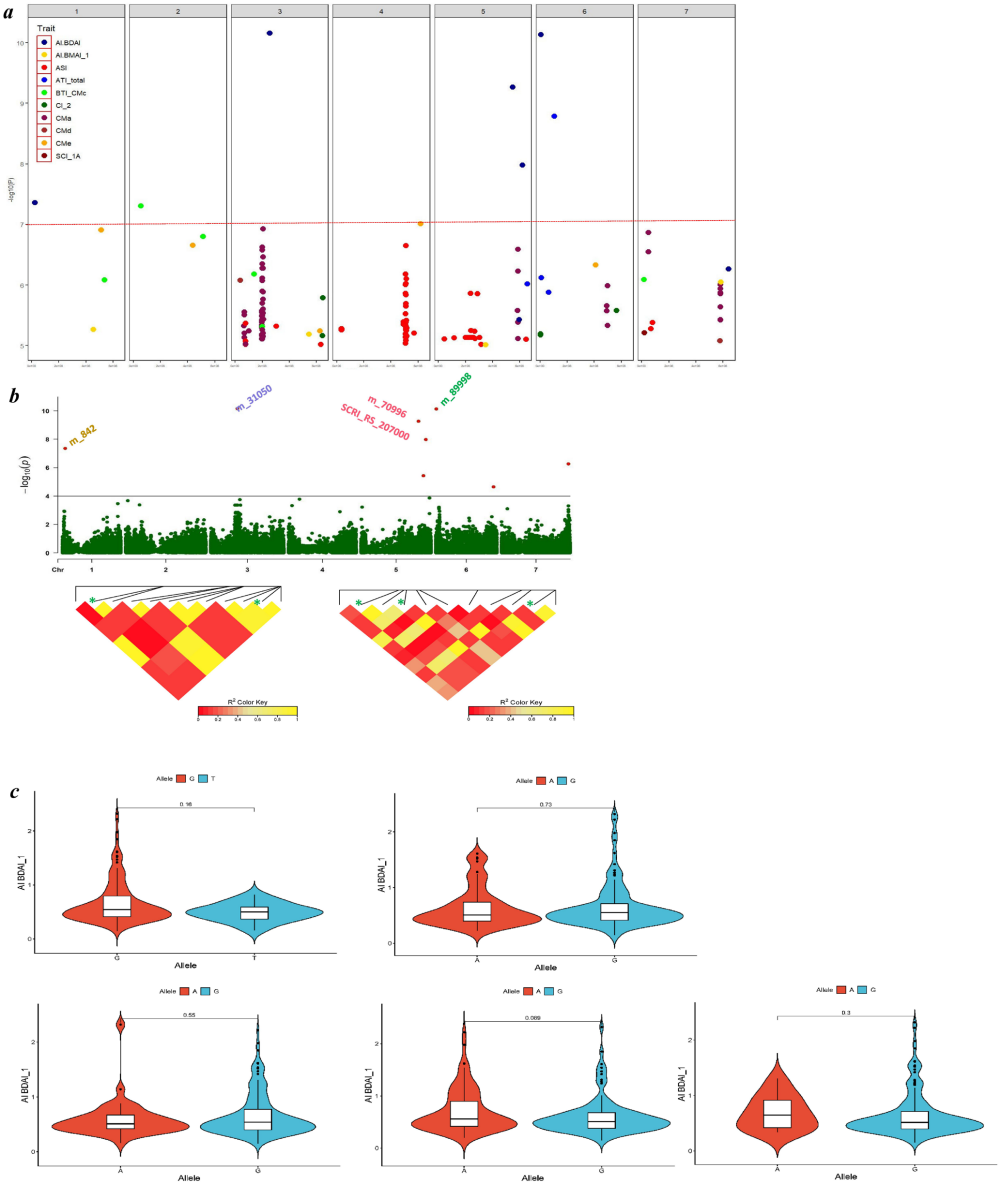
(a) A Manhattan plot shows the highly significant QTNs (− log_10_ ≥ 7) associated with ATIs. The *x*‐axis shows the chromosomes and the SNP order. The *y*‐axis shows the –log_10_ (*P*‐value) for each SNP marker. (b) Locus zoom on regional plot colocalization of highly associated QTNs inside LD heat map with AI.BDAI. (c) Allelic variation and their effects on the accessions at the natural genetic variant of single SNP.

To identify potential genes, we focused on the eight most essential QTNs (Figure 4a). These QTNs are found on all chromosomes except chromosome 7 and are related to several alternative transcript proteins. According to the physical position of QTNs, 63 putative candidate genes were detected within the defined interval of these QTNs (Table S3). Out of the high number of candidate genes, critical putative genes were found to be associated with ATIs. For example, the genes encoding protein family members, serine/threonine protein phosphatase, methionyl‐tRNA formyltransferase, and sulfur‐rich proteins (Table 2 and Table S3). The summary of the most promising candidate genes involved in ATI protein synthesis and/or accumulation is listed in Table 2.

**TABLE 2:** List of high‐confidence candidate genes with their physical position based on Morex (v3) genome pseudomolecule and annotation with gene ontology (GO) terms.

Gene name	Ch.	Start	End	Annotation	GO terms
*HORVU.MOREX.r3.1HG0039220.1*	1H	247,452,282	247,452,893	beta glucosidase 12	NA
*HORVU.MOREX.r3.2HG0179740.1*	2H	549,566,409	549,566,990	HAD superfamily, subfamily IIIB acid phosphatase	NA
*HORVU.MOREX.r3.3HG0261980.1*	3H	256,256,165	256,270,547	Serine/threonine‐protein phosphatase	GO:0004721, GO:0006470, GO:0016787, GO:0046872
*HORVU.MOREX.r3.3HG0262030.1*	3H	257,354,514	257,356,746	Serine/threonine‐protein phosphatase 7 long form‐like protein	NA
*HORVU.MOREX.r2.4HG0346780.1*	4H	618,496,587	618,497,660	Matrix metalloproteinase	GO:0004222, GO:0006508, GO:0008233, GO:0008237, GO:0008270, GO:0016787, GO:0031012, GO:0046872
*HORVU.MOREX.r2.4HG0346850.1*	4H	618,560,625	618,561,014	Serine/threonine protein phosphatase 7 long form isogeny	GO:0003824, GO:0004479, GO:0006413, GO:0009058, GO:0016740, GO:0016742, GO:0071951
*HORVU.MOREX.r2.4HG0346880.1*	4H	618,572,569	618,573,013	Methionyl‐tRNA formyltransferase	GO:0003824, GO:0004479, GO:0006413, GO:0009058, GO:0016740, GO:0016742, GO:0071951
*HORVU.MOREX.r2.4HG0346900.1*	4H	618,576,479	618,578,776	Methionyl‐tRNA formyltransferase	GO:0003824, GO:0004479, GO:0006413, GO:0009058, GO:0016740, GO:0016742, GO:0071951
*HORVU.MOREX.r2.4HG0346930.1*	4H	618,626,562	618,630,461	Phosphatidylserine decarboxylase proenzyme	GO:0004609, GO:0005739, GO:0005743, GO:0006629, GO:0006646, GO:0008654, GO:0016020, GO:0016021, GO:0016540, GO:0016829, GO:0016831, GO:0031305
*HORVU.MOREX.r2.4HG0347000.1*	4H	618,681,810	618,683,996	Nucellin‐like aspartic protease	GO:0004190, GO:0006508, GO:0008233, GO:0016787
*HORVU.MOREX.r3.5HG0520210.1*	5H	549,852,946	549,857,612	Aminopeptidase	GO:0004177, GO:0006508, GO:0016020, GO:0016021
*HORVU.MOREX.r2.5HG0430950.1*	5H	555,803,805	555,805,538	P‐loop containing nucleoside triphosphate hydrolases superfamily protein	GO:0000049, GO:0000166, GO:0003723, GO:0004812, GO:0004825, GO:0005524, GO:0005737, GO:0006412, GO:0006418, GO:0006431, GO:0016874, GO:0046872
*HORVU.MOREX.r2.5HG0430960.1*	5H	555,860,372	555,862,165	Pentatricopeptide repeat‐containing protein	GO:0008270
*HORVU.MOREX.r2.5HG0430980.1*	5H	555,877,795	555,879,957	Pentatricopeptide repeat‐containing protein	GO:0000139, GO:0001666, GO:0005515, GO:0005783, GO:0005789, GO:0005794, GO:0006629, GO:0007568, GO:0008202, GO:0008203, GO:0012507, GO:0015485, GO:0016020, GO:0016021, GO:0019217, GO:0031410, GO:0032868, GO:0032933, GO:0032991, GO:0042304, GO:0043231, GO:0044877, GO:0045540, GO:0045541, GO:0045716, GO:0051082
*HORVU.MOREX.r3.6HG0566970.1*	6H	104,409,764	104,413,944	Sulfurtransferase	GO:0004792, GO:0016740
*HORVU.MOREX.r3.6HG0567000.1*	6H	104,769,138	104,769,494	calpain‐type cysteine protease family	NA

**FIGURE 5:**
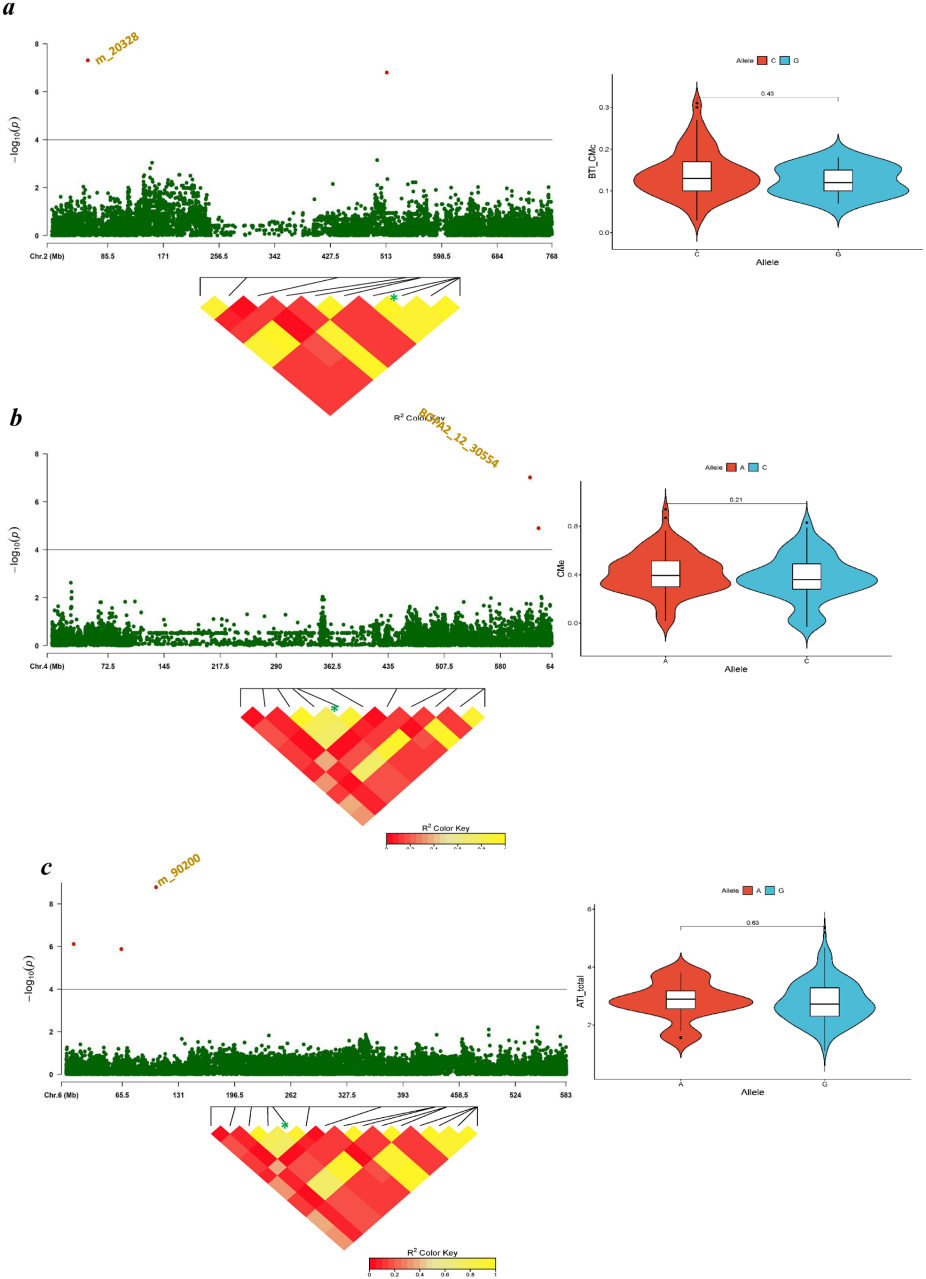
(a) Locus zoom on regional plot colocalization of highly associated QTNs inside LD heat map with BTI_CMc and the allelic variation and their effects on the accessions at the natural genetic variant of single SNP. (b) Locus zoom on regional plot colocalization of highly associated QTNs inside LD heat map with CMe and the allelic variation and their effects on the accessions at the natural genetic variant of single SNP. (c) Locus zoom on regional plot colocalization of highly associated QTNs inside LD heat map with ATI_total and the allelic variation and their effects on the accessions at the natural genetic variant of single SNP.

We narrowed down the regions of the highly significant QTNs to examine the allelic variation of these QTNs and explain the natural variation in ATI proteins among the accessions (Figures 4b,c and 5a–c). Locus zoom on five QTNs, which are colocalized inside LD regions and associated with AI. BDAI did not significantly impact the trait (Figure 4b,c). Figure 5a–c shows the locus zoom on the regions of QTNs associated with ATI. total, BTI_CMc, and CMe with their allelic variation at each SNP. Unfortunately, these SNPs showed slight differences among the alleles, but none showed significant allelic variation among the accessions carrying different alleles (Figures 4c and 5a–c).

## 4 Discussion

### 4.1 The Accumulation of Different Alpha‐Amylase/Trypsin Inhibitors in a Global Barley Collection Was Related to the Genotype's Biological Characteristics

In this study, we discovered the natural variation of ATIs and detected the underlying genetic factors controlling them, which is the first report in barley. Due to the importance of these traits, we performed this study under large field plots and under replication to check whether they are genetically controlled or affected by the environment. Despite the characterization of several ATIs in other cereals (Aslam et al. 2023; Geisslitz et al. 2023; Geisslitz et al. 2021; Geisslitz et al. 2022; Simonetti, Bosi, Negri, and Dinelli 2022), there is a crucial need to identify their presence in barley and to provide barley grain with reduced levels of ATIs for the production of food and beverages suitable for consumption by individuals with gastrointestinal sensitivities (Zevallos et al. 2017). In this context, assessing a global barley collection for ATIs is paramount for elucidating these proteins' genetic diversity and distribution across diverse barley varieties. Our novel study revealed a substantial phenotypic variation across the 184 worldwide accessions screened, ranging from 1.572 to 5.199 mg/g for total ATIs.

Furthermore, a comprehensive assessment of ATIs in a global barley collection can guide the selection of parental lines for breeding programs targeting specific markets, such as those requiring low‐ATI barley for gluten‐sensitive populations or high‐ATI barley for regions where pest resistance is critical. This approach ultimately contributes to the development of barley varieties that are both agronomically robust and tailored to meet the diverse needs of global consumers and farmers. Our results suggest that low‐ATI accessions such as the six‐rowed landraces HOR 2571, HOR 1684, HOR 1129, and the two‐rowed landraces HOR 4703 and HOR 6658 may be suitable for inclusion in breeding programs targeting gluten‐sensitive consumers.

In wheat, ATIs can stimulate the innate immune system in laboratory conditions and perhaps contribute to inflammation in the intestines (Zevallos et al. 2017). The studies demonstrated that consuming them caused a slight increase in the number of immune cells in the intestines and their activation, as well as the production of substances that cause inflammation, primarily observed in the colon, followed by the ileum and then the duodenum (Juhasz et al. 2018; Zevallos et al. 2017). A study revealed that individuals in modern countries typically consume around 15–30 g of gluten per day, comparable to around 0.5–1.5 g of ATIs, and gluten‐containing cereals have by far the highest concentrations of ATIs that activate TLR4 (Zevallos et al. 2017). Furthermore, the ATIs derived from wheat and other grains containing gluten, such as barley, stimulate innate immune responses in the gut by activating TLR4. Hence, consuming ATIs stimulates innate immune cells in pre‐existing low‐level inflammation in the small intestine and colon.

Zevallos et al. (2017) found that older wheat varieties, such as Emmer or Einkorn, had lower levels of bioactivity from ATI than modern hexaploid wheat. The contemporary wheat varieties were revealed to be the primary activators of TLR4 and had a high resistance to intestinal proteolysis. Interestingly, our results are in the same trend, where we found the ATIs were less prevalent in landraces than in breeding lines and hybrid lines. These results suggest the potential utility of Genebanks' landraces and ancient genotypes for reducing ATI levels in future breeding programs to improve flour quality, human nutrition, and health.

### 4.2 QTNs Associated With α‐Amylase/Trypsin Inhibitors Synthesis in a Global Barley Collection

Our novel study explores the power of GWAS to identify suitable quantitative nucleotide regions and candidate genes controlling Amylase Trypsin Inhibitors biosynthesis in a diverse barley collection. For this purpose, the FarmCPU model was applied using 127,022 polymorphic SNPs.

The high heritability reported for all treatments (0.70–0.97) showed the utility of the set for breeding programs aimed at improving barley's nutritional and health‐related traits. El Hassouni et al. (2021) reported lower heritability values ranging from 0.36 to 0.79 in wheat. At the same time, Bajgain et al. (2023) documented a heritability of 0.385 for ATI_total. Our results suggest a more significant potential for ATI improvement in barley grains via breeding.

The current study has identified a total of 444 QTNs with threshold‐log10 *p*‐values > FDR, and eight QTNs showed a highly significant association with ATI proteins, which are associated with a reduction in the biosynthesis of different α‐amylase/trypsin inhibitors. These findings can be used for marker‐assisted breeding and as a resource for improving the future barley‐based food, enhancing food quality and human health, especially for celiac disease and baker's asthma.

So far, genetic control studies of ATIs via GWAS in wheat have been documented (El Hassouni et al. 2021). Sequencing the wheat genome revealed many genes that produce seed‐borne immune‐responsive proteins, including ATI genes associated with celiac disease (Juhasz et al. 2018). In this regard, Bajgain et al. (2023) reported significant QTLs on chromosomes 2A (markers S2A_694955962 and S2A_694955959) and 7A (S7A_523147965) showing minor effects (4.9%–6.9% of the explained phenotypic variation). For their part, El Hassouni et al. (2021) reported three major QTLs on chromosome 3B (related to ATI 0.19‐like) and two on 6B (associated with ATI 0.28), explaining more than 68% of the genotypic variance. These authors also documented eight potential candidate genes explaining > 10% of the genotypic variance for ATI 0.19‐like (*TraesCS3B02G111100*, *TraesCS3B02G111200*, and *TraesCS3B02G294800*), ATI CM1 (*TraesCS1A02G048700*), ATI CM2 (*TraesCS7B02G072000*, *TraesCS1D02G163900*, and *TraesCS1D02G164000*), and total ATI (*TraesCS3B02G170800*).

In our study, we identified 63 putative candidate genes, of which there are critical putative genes annotated as Serine/threonine protein phosphatase, methionyl‐tRNA formyltransferase, and sulfur‐rich protein. We found 16 genes highly candidate for ATIs, but none were encoded as ATIs found on chromosomes 3 and 6 in wheat (Juhasz et al. 2018). Most of these genes were underlying AI. BDAI is annotated as a Serine/threonine protein phosphatase, such as *HORVU.MOREX.r3.3HG0261980* and *HORVU.MOREX.r2.4HG0346850*, while *HORVU.MOREX.r3.6HG0566970*, which encoded Sulfurtransferase, was the potential candidate gene underlying total ATI in barley grains. In plants, the serine/threonine protein phosphatases type 2A (*StPP2A*) participate in several physiological processes, serving crucial functions in developmental programs, stress responses, and hormone signaling (Muniz Garcia et al. 2022), which belong to the phosphoprotein phosphatase (PPP) family. Juhasz et al. (2018) discovered genes belonging to the prolamin superfamily and genes encoding members of the non‐prolamin allergen protein family, which are connected with ATIs linked to celiac disease and baker's asthma. They also found that protein fractions linked mainly to celiac disease were reduced during wheat seed storage under low temperatures. Still, this condition enhanced the concentration of protein families associated with WDEIA or baker's asthma, such as nsLTPs, ATIs, and hydrolases. A recent investigation of the genomes of current hexaploid wheat compared to ancient diploid wheat has shown a significant diversity of 19 ATI species encoded in the hexaploid wheat, whereas the diploid Einkorn species either have very few or no genes expressing ATI (Zevallos et al. 2017). In wheat, ATI gene expression was high primarily in transfer cells and starchy endosperm. It was associated with celiac disease and baker's asthma, which was attributed to a response to low temperature (Juhasz et al. 2018). In barley, based on the latest version of genome sequencing and annotation, no gene encodes ATI, and the gene expression of the candidate genes was not clear enough to build a hypothesis. Therefore, more studies are necessary to elucidate the genetic structure of ATI proteins in greater detail.

## 5 Conclusions

In conclusion, we observed significant natural diversity in the population's quantity and composition of the ATI proteins. The high heritability values observed for ATI indicate that these proteins are under substantial genetic control and have the potential to be effectively utilized in future efforts to improve the quality of barley‐based food products. This first report establishes the genetic factors determining the ATI accumulation in barley accessions using GWAS. The findings of this study underscore the significance of our research, as it is the pioneering investigation to elucidate the phenotypic diversity and genetic determinants underlying the accumulation of ATIs in a global barley collection. LD breaks occur due to recombination in the population, which helps find the significant genomic area, detecting many minor effects of QTNs. The identified QTNs/candidate genes shed light on the genetic improvement of barley genotypes for human health and nutrition. Notwithstanding the efforts undertaken, human clinical trial investigations are essential to substantiate the ATI's impact as a notable allergen and assess gene expression. Ultimately, using genetic techniques to reduce the level of ATI in barley grains would take considerable time. To expedite the process, including landrace barley (characterized by low ATIs) in the food supply chain for milling would offer a more efficient alternative process and reduce the effect of ATIs on human health.

## Author Contributions

**Dalia Z. Alomari:** conceptualization (equal), data curation (equal), formal analysis (equal), investigation (equal), methodology (equal), software (equal), writing – original draft (equal), writing – review and editing (equal). **Samar G. Thabet:** data curation (equal), formal analysis (equal), investigation (equal), software (equal), visualization (equal). **Mashael D. Alqahtani:** funding acquisition (equal), visualization (equal), writing – original draft (equal). **Matías Schierenbeck:** data curation (equal), formal analysis (equal), software (equal). **Sarah Joestl:** investigation (equal), methodology (equal), visualization (equal). **Sabrina Geisslitz:** methodology (equal). **Katharina A. Scherf:** conceptualization (equal), methodology (equal), supervision (equal). **Andreas Börner:** conceptualization (equal), project administration (equal), resources (equal), supervision (equal). **Ahmad M. Alqudah:** conceptualization (equal), data curation (equal), formal analysis (equal), investigation (equal), methodology (equal), supervision (equal), validation (equal), visualization (equal), writing – original draft (equal), writing – review and editing (equal).

## Ethics Statement

The authors have nothing to report.

## Conflicts of Interest

The authors declare no conflicts of interest.

## Supporting information


**Figure S1:** Accumulation of different Amylase trypsin inhibitors (ATIs) in 184 spring barley genotypes.
**Figure S2:** Manhattan plots showing significant marker traits association and Quantile‐Quantile (Q‐Q) for Farm‐CPU model for (a) AI.BDAI and (b) AI.BMAI in 184 spring barley genotypes (*p* < 0.0001; −log_10_ > FDR). Red line represents the expected values.
**Figure S3:** Manhattan plots showing significant marker traits association and Quantile‐Quantile (Q‐Q) for Farm‐CPU model for (a) ASI and (b) ATI_total in 184 spring barley genotypes (*p* < 0.0001; −log10 > FDR). Red line represents the expected values.
**Figure S4:** Manhattan plots showing significant marker traits association and Quantile‐Quantile (Q‐Q) for Farm‐CPU model for (a) BTI_CMc and (b) CI_2 in 184 spring barley genotypes (*p* < 0.0001; −log10 > FDR). Red line represents the expected values.
**Figure S5:** Manhattan plots showing significant marker traits association and Quantile‐Quantile (Q‐Q) for Farm‐CPU model for (a) CMc and (b) CMd in 184 spring barley genotypes (*p* < 0.0001; −log10 > FDR). Red line represents the expected values.
**Figure S6:** Manhattan plots showing significant marker traits association and Quantile‐Quantile (Q‐Q) for Farm‐CPU model for (a) CMe and (b) SCI_1A in 184 spring barley genotypes (*p* < 0.0001; −log10 > FDR). Red line represents the expected values.

**Table S1:** Summary statistics of different Amylase trypsin inhibitors (ATIs) in 184 spring barley accessions.

## Data Availability

The data used to support the findings of this study is included in the article.
