# Supplementary material for: The Genetic Factors Controlling the Accumulation of Amylase/Trypsin Inhibitors (ATIs) in Barley for Enhancing Human Nutrition and Health
Source: Food Sci Nutr. 2026 Jan 10;14(1):e70990. doi: 10.1002/fsn3.70990 (PMC12789939; doi:10.1002/fsn3.70990)
Supplement: Supplementary file 1 — Figure S1: Accumulation of different Amylase trypsin inhibitors (ATIs) in 184 spring barley genotypes. Figure S2: Manhattan plots showing significant marker traits association and Quantile‐Quantile (Q‐Q) for Farm‐CPU model for (a) AI.BDAI and (b) AI.BMAI in 184 spring barley genotypes (p < 0.0001; −log10 > FDR). Red line represents the expected values. Figure S3: Manhattan plots showing significant marker traits association and Quantile‐Quantile (Q‐Q) for Farm‐CPU model for (a) ASI and (b) ATI_total in 184 spring barley genotypes (p < 0.0001; −log10 > FDR). Red line represents the expected values. Figure S4: Manhattan plots showing significant marker traits association and Quantile‐Quantile (Q‐Q) for Farm‐CPU model for (a) BTI_CMc and (b) CI_2 in 184 spring barley genotypes (p < 0.0001; −log10 > FDR). Red line represents the expected values. Figure S5: Manhattan plots showing significant marker traits association and Quantile‐Quantile (Q‐Q) for Farm‐CPU model for (a) CMc and (b) CMd in 184 spring barley genotypes (p < 0.0001; −log10 > FDR). Red line represents the expected values. Figure S6: Manhattan plots showing significant marker traits association and Quantile‐Quantile (Q‐Q) for Farm‐CPU model for (a) CMe and (b) SCI_1A in 184 spring barley genotypes (p < 0.0001; −log10 > FDR). Red line represents the expected values. [file FSN3-14-e70990-s002.pptx]

## Slide 1
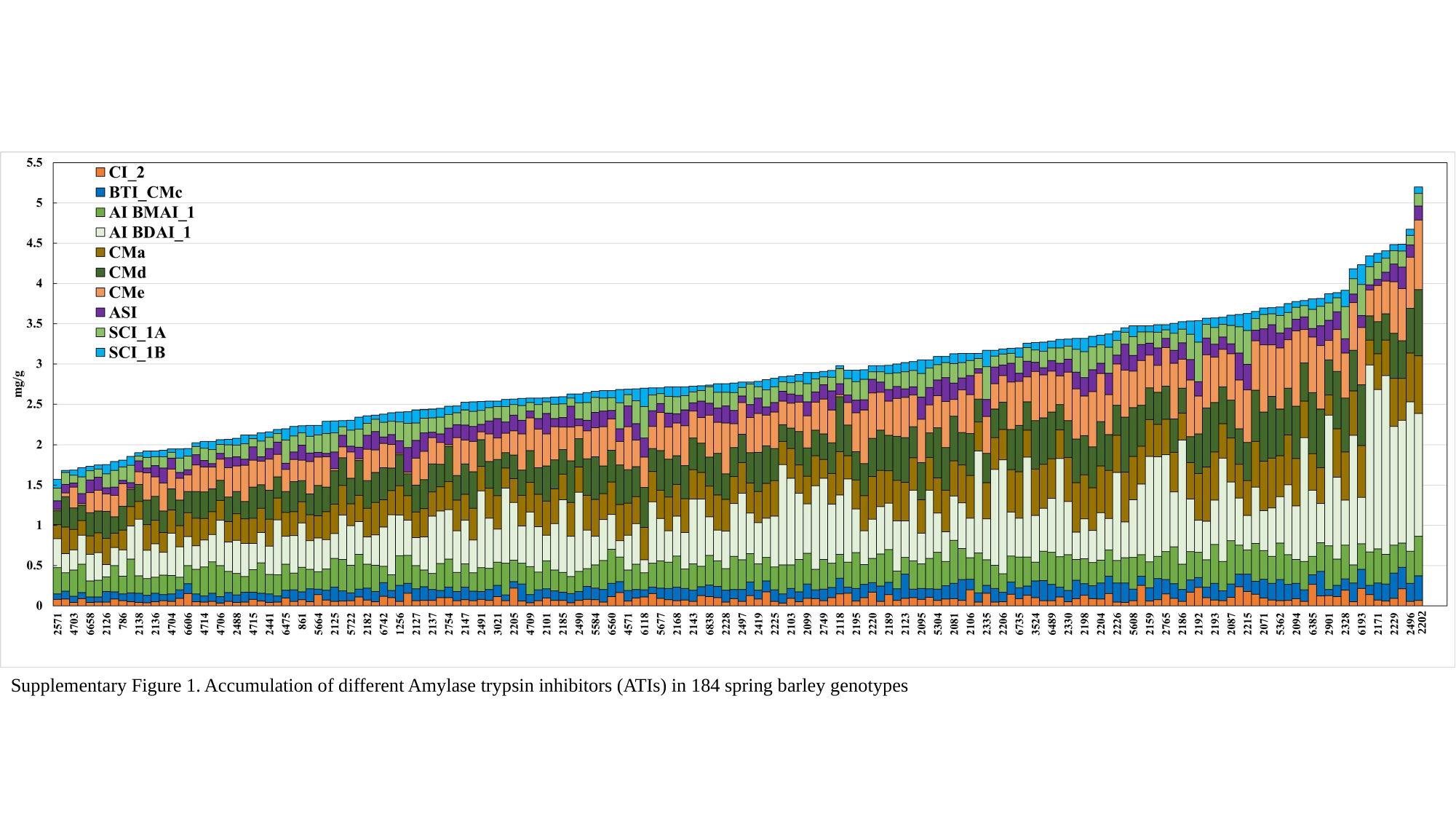

2202
Supplementary Figure 1. Accumulation of different Amylase trypsin inhibitors (ATIs) in 184 spring barley genotypes

## Slide 2
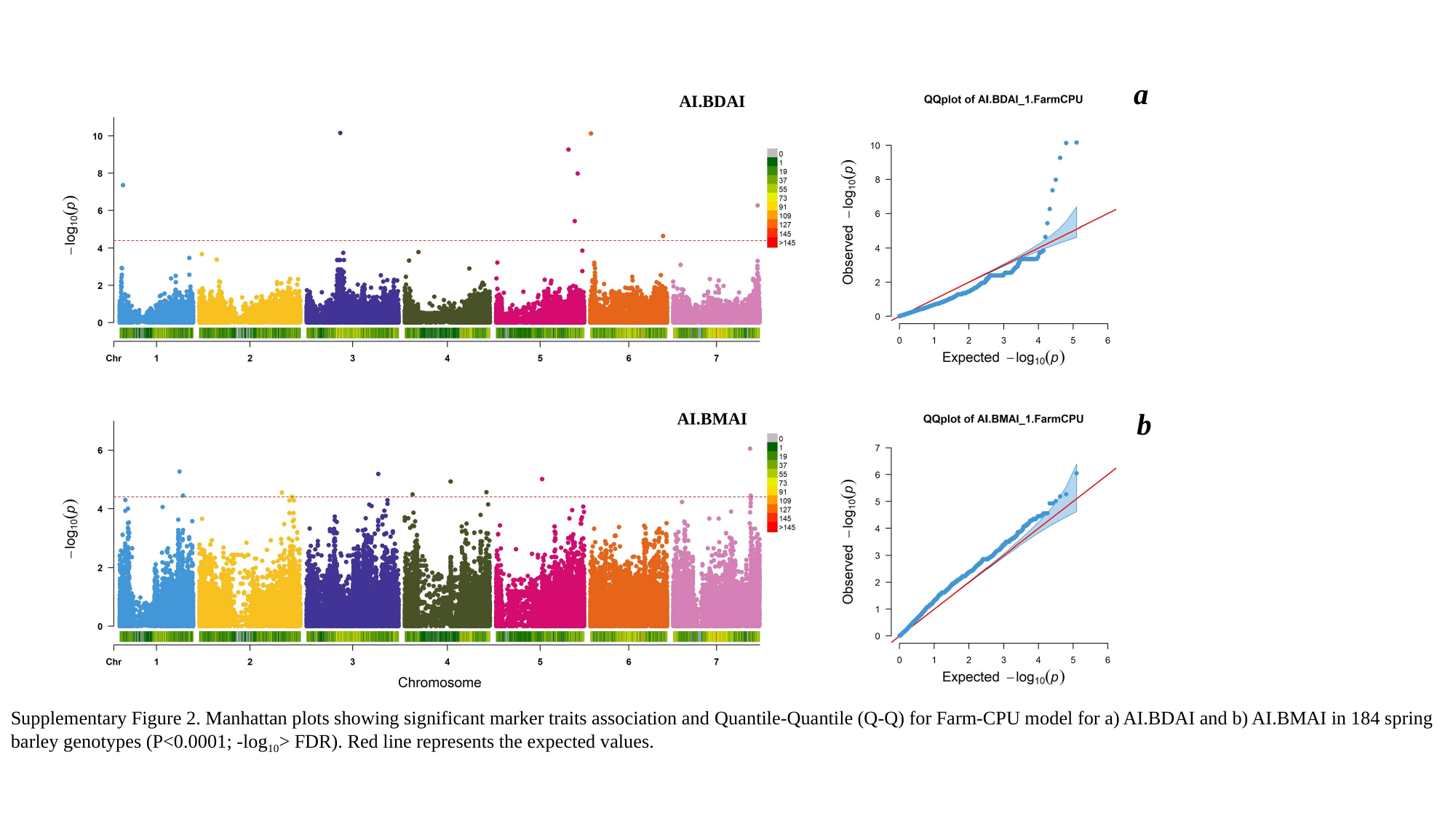

AI.BDAI
AI.BMAI
a
b
Supplementary Figure 2. Manhattan plots showing significant marker traits association and Quantile-Quantile (Q-Q) for Farm-CPU model for a) AI.BDAI and b) AI.BMAI in 184 spring barley genotypes (P<0.0001; -log10> FDR). Red line represents the expected values.

## Slide 3
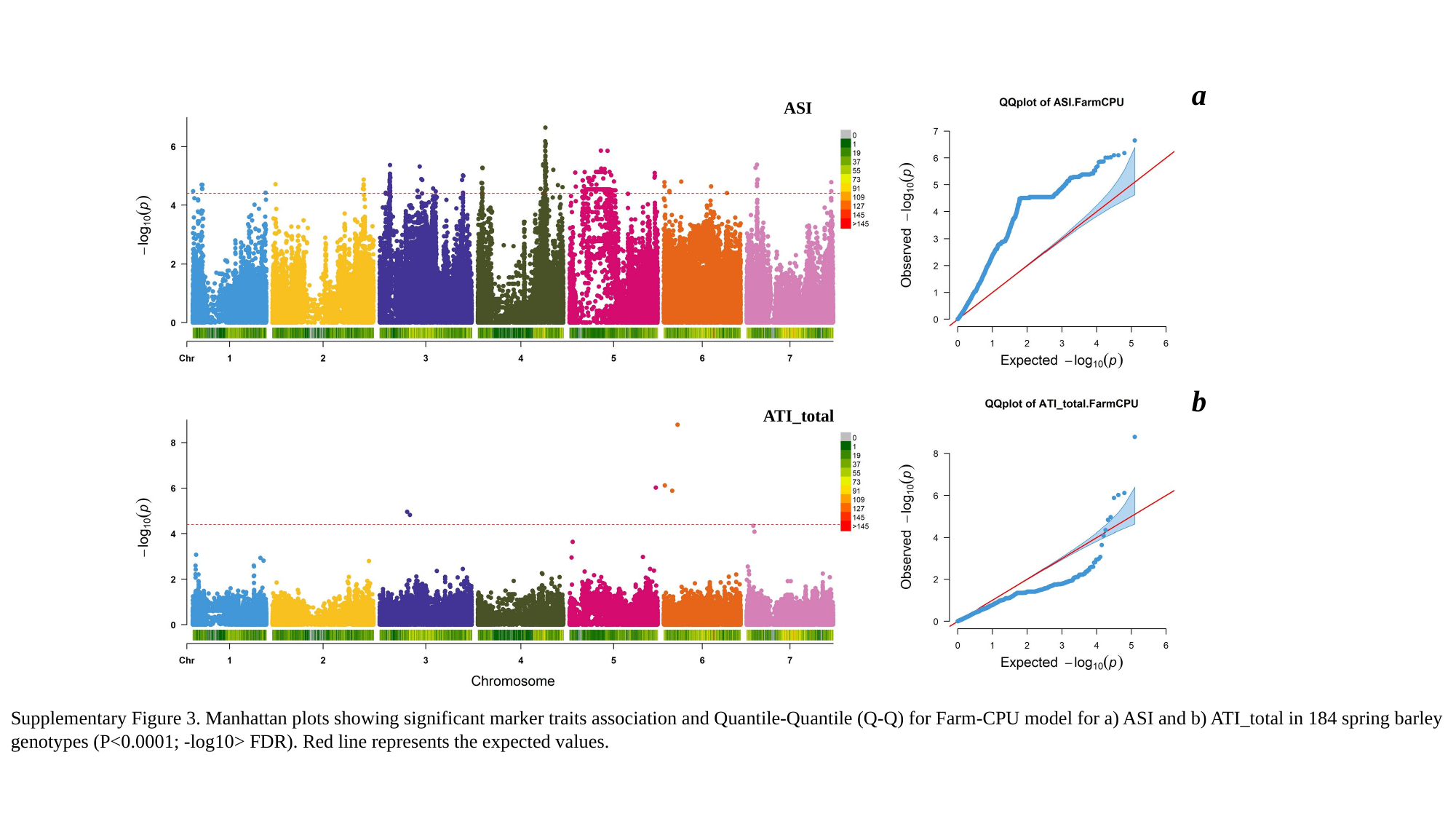

ASI
ATI_total
a
b
Supplementary Figure 3. Manhattan plots showing significant marker traits association and Quantile-Quantile (Q-Q) for Farm-CPU model for a) ASI and b) ATI_total in 184 spring barley genotypes (P<0.0001; -log10> FDR). Red line represents the expected values.

## Slide 4
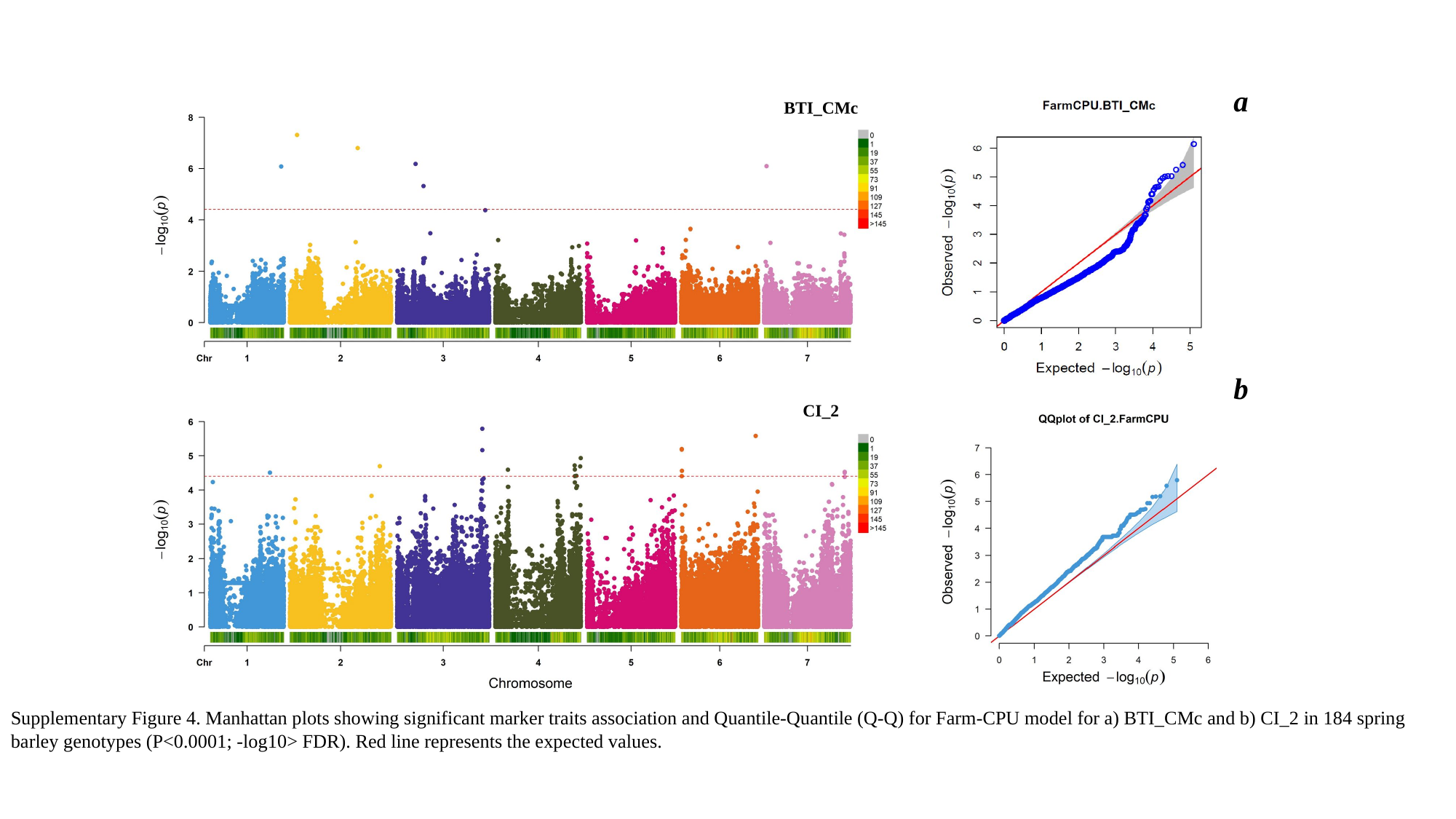

a
BTI_CMc
b
CI_2
Supplementary Figure 4. Manhattan plots showing significant marker traits association and Quantile-Quantile (Q-Q) for Farm-CPU model for a) BTI_CMc and b) CI_2 in 184 spring barley genotypes (P<0.0001; -log10> FDR). Red line represents the expected values.

## Slide 5
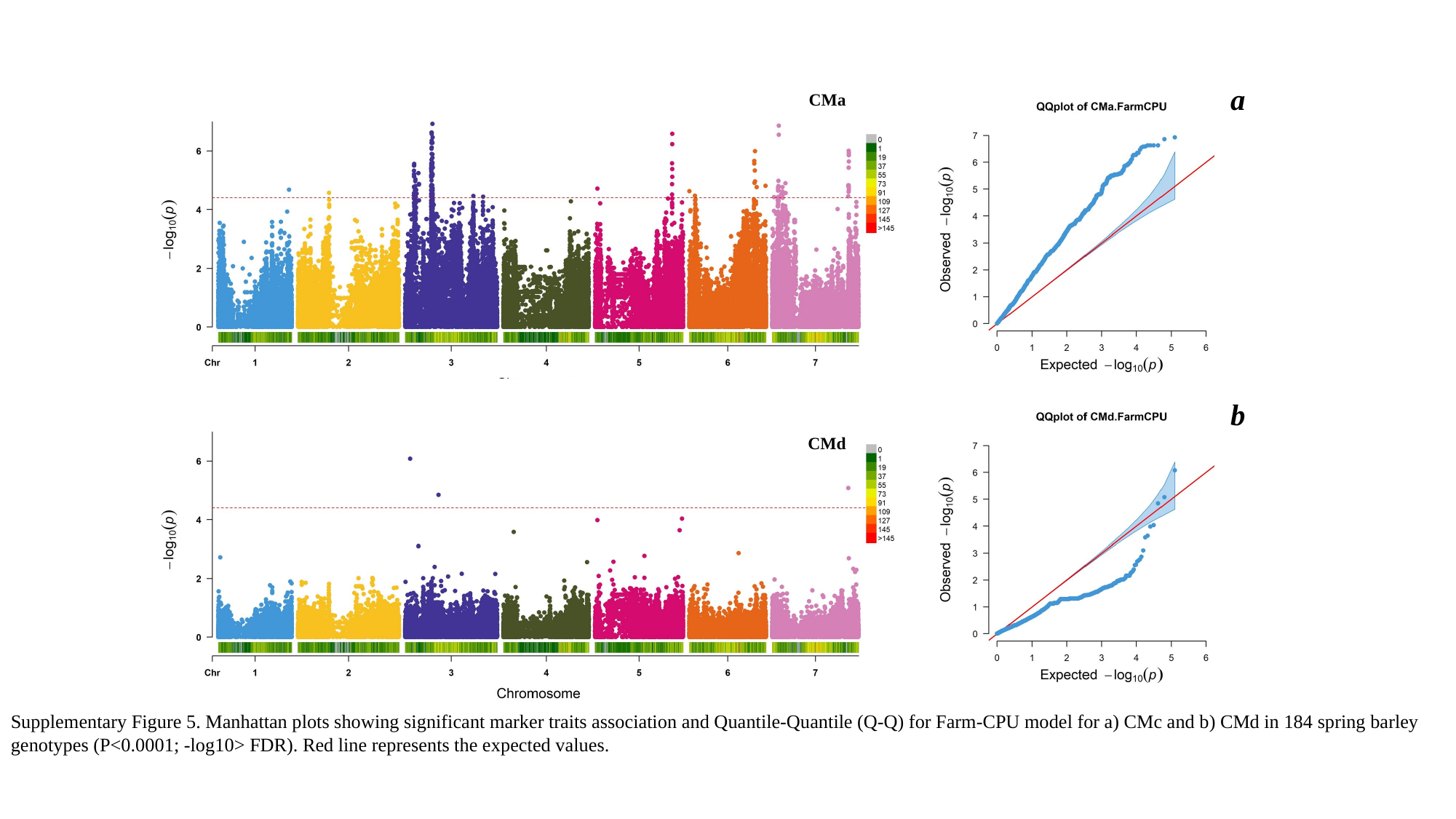

CMa
CMd
a
b
Supplementary Figure 5. Manhattan plots showing significant marker traits association and Quantile-Quantile (Q-Q) for Farm-CPU model for a) CMc and b) CMd in 184 spring barley genotypes (P<0.0001; -log10> FDR). Red line represents the expected values.

## Slide 6
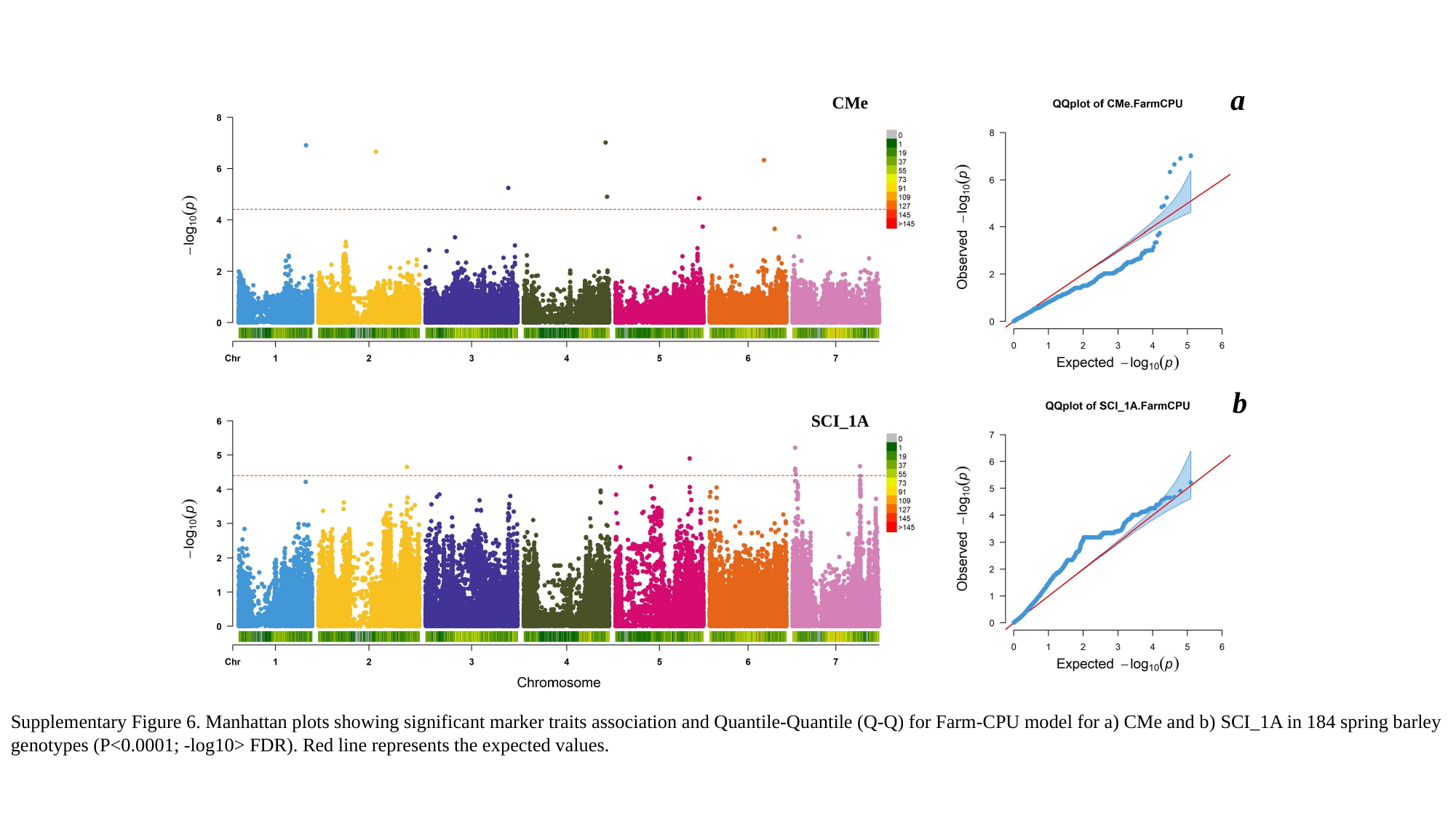

CMe
SCI_1A
a
b
Supplementary Figure 6. Manhattan plots showing significant marker traits association and Quantile-Quantile (Q-Q) for Farm-CPU model for a) CMe and b) SCI_1A in 184 spring barley genotypes (P<0.0001; -log10> FDR). Red line represents the expected values.
